# P-1273. Molecular Epidemiology of Antimalarial Drug Resistance in Vivax Malaria Clinical Samples from the Southwestern Region of India

**DOI:** 10.1093/ofid/ofaf695.1463

**Published:** 2026-01-11

**Authors:** Vishnu Teja Nallapati, Kavitha Saravu, Prashanth Bhat, Sushma Belurkar, Manjunath Hande

**Affiliations:** Kasturba Medical College, Manipal, Manipal Academy of Higher Education, Manipal, Manipal, Karnataka, India; Kasturba Medical College, Manipal, Karnataka, India, Manipal, Karnataka, India; Department of Health and Family Welfare, Government of Karnataka, Udupi District, Udupi, Karnataka, India; Kasturba Medical College, Manipal, Manipal Academy of Higher Education, Manipal, Manipal, Karnataka, India; Kasturba Medical College, Manipal, Manipal Academy of Higher Education, Manipal, Manipal, Karnataka, India

## Abstract

**Background:**

*Plasmodium vivax* is a major cause of malaria in India, especially in the southwest. Chloroquine (CQ) followed by 14-day Primaquine is the recommended treatment for vivax malaria in India. However, antimalarial drug resistance poses a significant threat to effective treatments. Therefore, this study investigates the molecular epidemiology of drug resistance in vivax malaria clinical samples from southwestern India.

**Fig 1.**
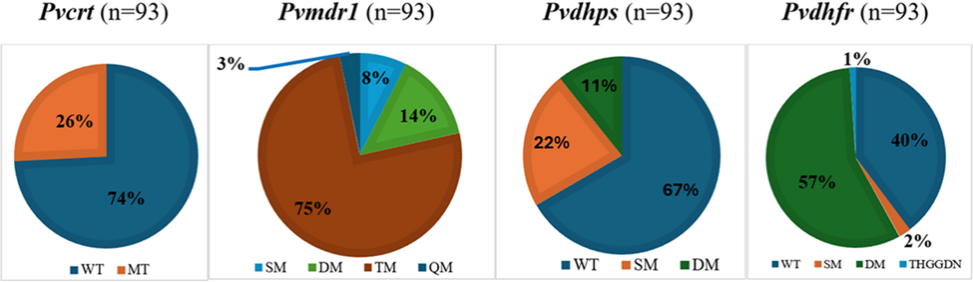
Prevalence of Pvcrt-o, Pvmdr1, Pvdhfr, and Pvdhps haplotypes among 93 P. vivax samples WT – wild type; SM – Single mutant; DM – Double mutant, TM – Triple mutant, QM – Quadruple mutant

**Methods:**

A total of 93 *P. vivax* malaria samples were collected from symptomatic adult patients hailing from two districts (i.e., Udupi and Mangalore) of the southwestern region of India between June 2021 and 2023. All samples were amplified and sequenced for antimalarial drug resistance markers, including *Pvcrt-o, Pvmdr1, Pvdhf*r, and *Pvdhps* genes using Sanger sequencing. Genetic polymorphisms and haplotypes were identified and analyzed to assess the prevalence of resistance-associated mutations.

**Results:**

In *Pvcrt-o*, the K10 “AAG” insertion was detected in 26% of clinical samples, with only one case among those harboring this mutation showing persistent fever after treatment with CQ. For *Pvmdr1*, mutations M908L, T958M, Y976F, and F1076L were detected in 100%, 86.02%, 2.15%, and 84.94% of samples, respectively. In *Pvdhps*, mutations A383G and A553G occurred in 26.88% and 13.97% of samples, respectively. Meanwhile, *Pvdhfr* exhibited the S58R and S117N mutations in 59.13% and 56.98% of samples, with the double mutant (S58R+S117N) emerging as the most prevalent haplotype (Fig. 1).

**Conclusion:**

While mutations in *P. vivax* clinical samples do not indicate complete CQ resistance, they raise concerns about reduced drug efficacy. Detection of the Y976F mutation, known for chloroquine resistance in vitro, in one sample warrants continued monitoring. Furthermore, the high prevalence of S58R and S117N mutations in *Pvdhfr* (56.98%) likely results from selective pressure due to widespread ACT AS+SP use for *P. falciparum* malaria. Although CQ treatment showed minimal clinical failure in P. vivax with follow-up data, these genetic markers highlight the need for ongoing molecular surveillance and targeted antimalarial strategies to maintain efficacy.

**Disclosures:**

All Authors: No reported disclosures

